# Improving the efficiency of cadmium sulfide-sensitized titanium dioxide/indium tin oxide glass photoelectrodes using silver sulfide as an energy barrier layer and a light absorber

**DOI:** 10.1186/1556-276X-9-605

**Published:** 2014-11-07

**Authors:** Chong Chen, Yong Zhai, Chunxi Li, Fumin Li

**Affiliations:** 1 Henan Key Laboratory of Photovoltaic Materials, Henan University, Kaifeng 475004, People's Republic of China; 2 School of Physics and Electronics, Henan University, Kaifeng 475004, People's Republic of China

**Keywords:** Silver sulfide nanocrystals, Titanium dioxide, Photoelectrodes, Efficiency, Recombination

## Abstract

Cadmium sulfide (CdS) and silver sulfide (Ag_2_S) nanocrystals are deposited on the titanium dioxide (TiO_2_) nanocrystalline film on indium tin oxide (ITO) substrate to prepare CdS/Ag_2_S/TiO_2_/ITO photoelectrodes through a new method known as the molecular precursor decomposition method. The Ag_2_S is interposed between the TiO_2_ nanocrystal film and CdS nanocrystals as an energy barrier layer and a light absorber. As a consequence, the energy conversion efficiency of the CdS/Ag_2_S/TiO_2_/ITO electrodes is significantly improved. Under AM 1.5 G sunlight irradiation, the maximum efficiency achieved for the CdS(4)/Ag_2_S/TiO_2_/ITO electrode is 3.46%, corresponding to an increase of about 150% as compared to the CdS(4)/TiO_2_/ITO electrode without the Ag_2_S layer. Our experimental results show that the improved efficiency is mainly due to the formation of Ag_2_S layer that may increase the light absorbance and reduce the recombination of photogenerated electrons with redox ions from the electrolyte.

## Background

Dye-sensitized photoelectrodes consisting of a wide band gap semiconductor film and a dye form the basis of many applications in photocatalytic, optoelectronic, and photovoltaic devices [1-10]. In photovoltaic applications, the photoelectrodes are typically titanium dioxide (TiO_2_) films, which are sensitized by an organic or inorganic dye [7,9,11]. In dye-sensitized photoelectrodes, the dye plays an important role in light absorption and charge transfer. Compared with organic dyes, semiconductor nanocrystals (i.e., inorganic dyes) with their size-tunable absorption and high molar extinction coefficient [12,13] are superior in thermal and photochemical stability. Due to these advantages of semiconductor nanocrystals, theoretically, semiconductor nanocrystal-sensitized solar cells may have a maximum efficiency of 44%, which is much higher than that of organic dye-sensitized solar cells [14].

So far, various types of inorganic nanocrystals such as CdS [15-17], CdTe [15,16], CuInS_2_[18,19], Ag_2_S [20-24], and PbS [25,26] have been incorporated on TiO_2_ photoelectrodes as sensitizers to enhance the light absorption of the TiO_2_ photoelectrodes in the visible light region. Among single nanocrystal-sensitized TiO_2_ photoelectrodes, CdS-sensitized TiO_2_ photoelectrodes show a better photoelectric conversion performance. The efficiency of over 4% has been reported for CdS-sensitized TiO_2_ nanotube array photoelectrodes. However, it is still much lower than that of organic dye-sensitized TiO_2_ photoelectrodes [27-30]. The low efficiency is mostly caused by the serious charge recombination between the electrolyte and photoelectrodes [31]. Thus, to increase the conversion efficiency of the semiconductor nanocrystal-sensitized TiO_2_ photoelectrodes, considerable efforts have been made to suppress the charge recombination between the electrolyte and electrode. One common method for decreasing the charge recombination is to interpose an intermediate layer, such as a ZnS coating, between the inorganic nanocrystals and the electrolyte. Besides, another effective method, interposing an energy barrier layer between the TiO_2_ and electrolyte, has been recently reported. For example, a ZnO layer was deposited on the TiO_2_ photoelectrodes to significantly decrease the charge recombination in CdSe [32], CdS [17], and Ag_2_S [33] nanocrystal-sensitized TiO_2_ photoelectrodes. Similarly, a CuInS_2_ nanocrystal film was formed between the TiO_2_ photoelectrode and CdS to suppress the charge recombination in CuInS_2_-sensitized TiO_2_ photoelectrodes [19]. Among these reported nanocrystals, Ag_2_S has a narrow band gap of 0.9 to 1.05 eV and a larger absorption coefficient, which makes it an important material for photovoltaic application [33-35]. Furthermore, for these reported nanocrystals, the most commonly employed synthetic methods include solution synthesis [18,36,37], chemical bath deposition (CBD) [17,33], and successive ionic layer absorption and reaction (SILAR) [14]. For example, the Ag_2_S [20,33] and CdS [17,19] nanocrystals are commonly prepared by a CBD method.

**Figure 1:**
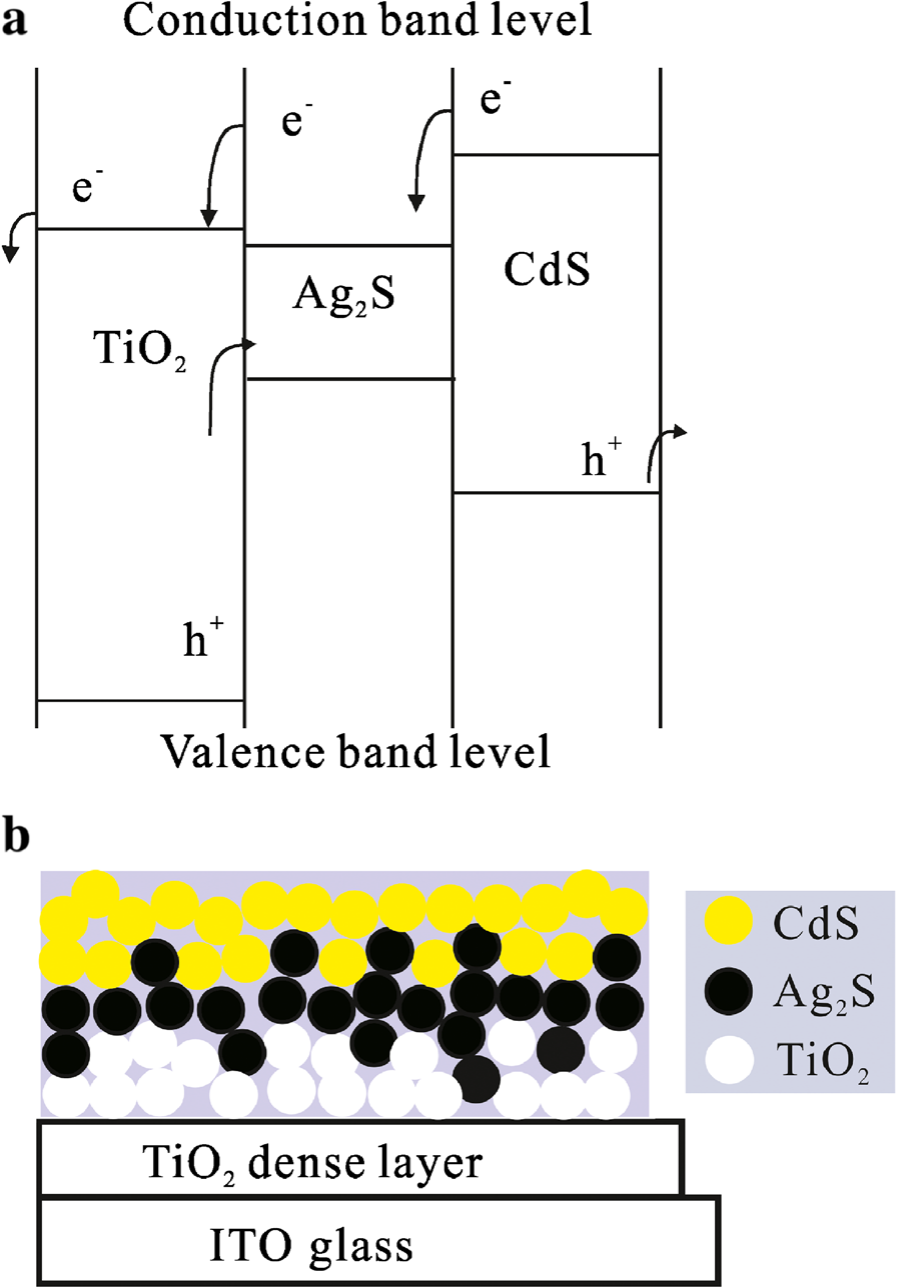
**Schematic diagram (a) and energy diagram (b) of the CdS/Ag**_**2**_**S/TiO**_**2**_**/ITO electrode.**

Unlike most of the previous studies, herein, both Ag_2_S and CdS nanocrystals were synthesized through a spin coating method of using a molecular-based precursor solution. In this study, we have successfully interposed an Ag_2_S nanocrystal film by the molecular precursor decomposition (MPD) method as an energy barrier layer between the TiO_2_ nanocrystal film on the transparent indium tin oxide (ITO) glass substrate (i.e., TiO_2_/ITO photoelectrode) and CdS nanocrystals. The main advantage of the MPD method is that it allows a greater deposition amount of nanocrystals and avoids the detachment of nanocrystals from the TiO_2_ photoelectrodes compared to CBD or SILAR method. The schematic diagram and energy diagram of prepared CdS/Ag_2_S/TiO_2_/ITO photoelectrode are shown in Figure 1. Our results demonstrated that the Ag_2_S nanocrystal film not only increases the light absorption of the CdS-sensitized TiO_2_/ITO photoelectrode but also effectively decreases the charge recombination between the TiO_2_ and electrolyte, which significantly enhanced the energy conversion efficiency of CdS-sensitized TiO_2_/ITO photoelectrode.

## Methods

### Materials

Cadmium chloride (CdCl_2_, 98.0%) was purchased from Kanto Chemical Co. Inc. Titanium tetrachloride (TiCl_4_, 99.995%), nitric acid (HNO_3_, 70%), hydrochloric acid (HCl, 37%), ethyl cellulose (CAS 9004-57-3), silver acetate (AgOAc, 99%), thiourea (≥99.0%), terpineol (≥96%), Ti(OCH_2_CH_2_CH_2_CH_3_)_4_ (Ti(OBu)_4_, 97%), 1-butylamine (99.5%), and 1-propionic acid (≥99.5%) were purchased from Sigma-Aldrich (St. Louis, MO, USA). All the reagents were used without further purification. Indium tin oxide coated glass slides (ITO, ≤15 Ω/sq, Wuhu Token Sci. Co., Ltd., Wuhu, China) were cleaned by successive sonication in deionized water, acetone, and isopropyl alcohol and then dried with nitrogen gas.

### Formation of TiO_2_ nanocrystalline film on ITO substrate

First, a TiO_2_ dense layer was introduced on the cleaned ITO substrate by spin coating a TiO_2_ sol-gel precursor at 3,000 rpm for 60 s. The procedure for the preparation of TiO_2_ sol-gel has been reported previously [38]. Briefly, 10 ml Ti(OBu)_4_ was dissolved in 60 ml ethanol and stirred about 5 min at room temperature. After that, 5 ml acetyl acetone was added and stirring was continued for 15 min. Then, a solution composed of 30 ml ethanol, 10 ml deionized (DI) water, and 2 ml HCl with a density of 0.28 mol/l was added dropwise under vigorous stirring. The final mixture was stirred at room temperature for 2 h to obtain a TiO_2_ sol-gel precursor. The substrates were annealed at 450°C for 2 h in a muffle furnace.

Secondly, the TiO_2_ nanocrystalline film was deposited on the prepared TiO_2_ dense layer. The solution-processed nanocrystalline titanium (TiO_2_) film was prepared as follows. A total of 2 g of titanium nanoparticles (TiO_2_ P25, Degussa, Frankfurt, Germany) was initially dissolved in 100 mL HNO_3_ solution (0.1 mol/L) and stirred for 12 h at 200°C. Afterward, the obtained solution was centrifuged at 7,000 rpm for 3 min to collect the TiO_2_ nanoparticles. To remove the remaining water and acid, the obtained TiO_2_ nanoparticles were re-dispersed in DI water and then the mixture was centrifuged at 7,000 rpm for 3 min. This washing step was repeated three times. The final product was dried at room temperature to get dried TiO_2_ nanoparticles. After that, the TiO_2_ paste consisting of 11.6% dried TiO_2_ nanoparticles and 5% ethyl cellulose in terpineol was prepared, which was spin cast on the TiO_2_ dense layer at 2,000 rpm. Then, the samples were annealed at 500°C for 30 min in a muffle furnace to obtain the TiO_2_/ITO films.

### Synthesis of Ag_2_S and CdS nanocrystals

Ag_2_S nanocrystals were synthesized through a method of using a molecular-based precursor solution. First, AgOAc (0.1 mmol) and thiourea (0.2 mmol) were dissolved in a mixture of 1-butylamine (0.7 mL) and 1-propionic acid (45 μL) under a nitrogen atmosphere in a glove box (O_2_ < 0.1 ppm, H_2_O <0.1 ppm). The mixture was then stirred for 3 min; after which, the obtained Ag_2_S precursor solution was then spin cast onto the prepared TiO_2_/ITO substrates at 1,500 rpm for 30 s. The obtained films were calcined at 150°C for 10 min and then heated to 250°C and held 15 min at this temperature to obtain the Ag_2_S/TiO_2_/ITO films. The CdS nanocrystals were synthesized through the same process. Briefly, the prepared CdS precursor solution composed of 0.1 mmol CdCl_2_ and thiourea (0.3 mmol) was spin cast onto the prepared Ag_2_S/TiO_2_/ITO films at 1,500 rpm for 30 s and then the films were calcined. Such a spinning-drying cycle was repeated several times to increase the thickness of the CdS film. The CdS/Ag_2_S/TiO_2_/ITO film after *n* cycles of the CdS deposition was denoted as CdS(*n*)/Ag_2_S/TiO_2_/ITO. The schematic diagram of CdS(*n*)/Ag_2_S/TiO_2_/ITO electrode is shown in Figure 1b. For comparison, the CdS(*n*)/TiO_2_/ITO films without Ag_2_S were also fabricated by the same process.

### Characterization and photovoltaic measurements

The structural and optical analyses of the prepared films were studied by X-ray diffractometer (XRD; DX-2500, Dandong Fangyuan Instrument Co., Ltd., Dandong, China) and UV-VIS-NIR spectrophotometer (UV-2550, Shimadzu Corporation, Kyoto, Japan), respectively. The surface morphologies were observed by scanning electron microscopy with energy dispersive X-ray analysis (EDX) (SEM, JSM-7001 F, Japan Electron Optics Laboratory Co., Ltd., Tokyo, Japan). Photoelectrochemical experiments were performed using an electrochemical workstation (CHI660E, Shanghai Chenhua Instruments Co., Ltd., Shanghai, China) using a three-electrode configuration with the as-prepared samples as working electrode, a Pt foil counter electrode, and a saturated Ag/AgCl reference electrode, and devices were illuminated with a calibrated AM 1.5 solar light simulator (Newport Inc., Irvine, CA, USA) operating at an intensity of 100 mW cm^−2^. The light intensity was calibrated with a monocrystalline Si reference cell. The electrolyte was 1.0 M Na_2_S aqueous solution. The photocurrent responses of the working electrodes with a surface area of 0.5 cm^−2^ were recorded during a voltage sweep from −1.4 to 0.2 V.

## Results and discussion

**Figure 2:**
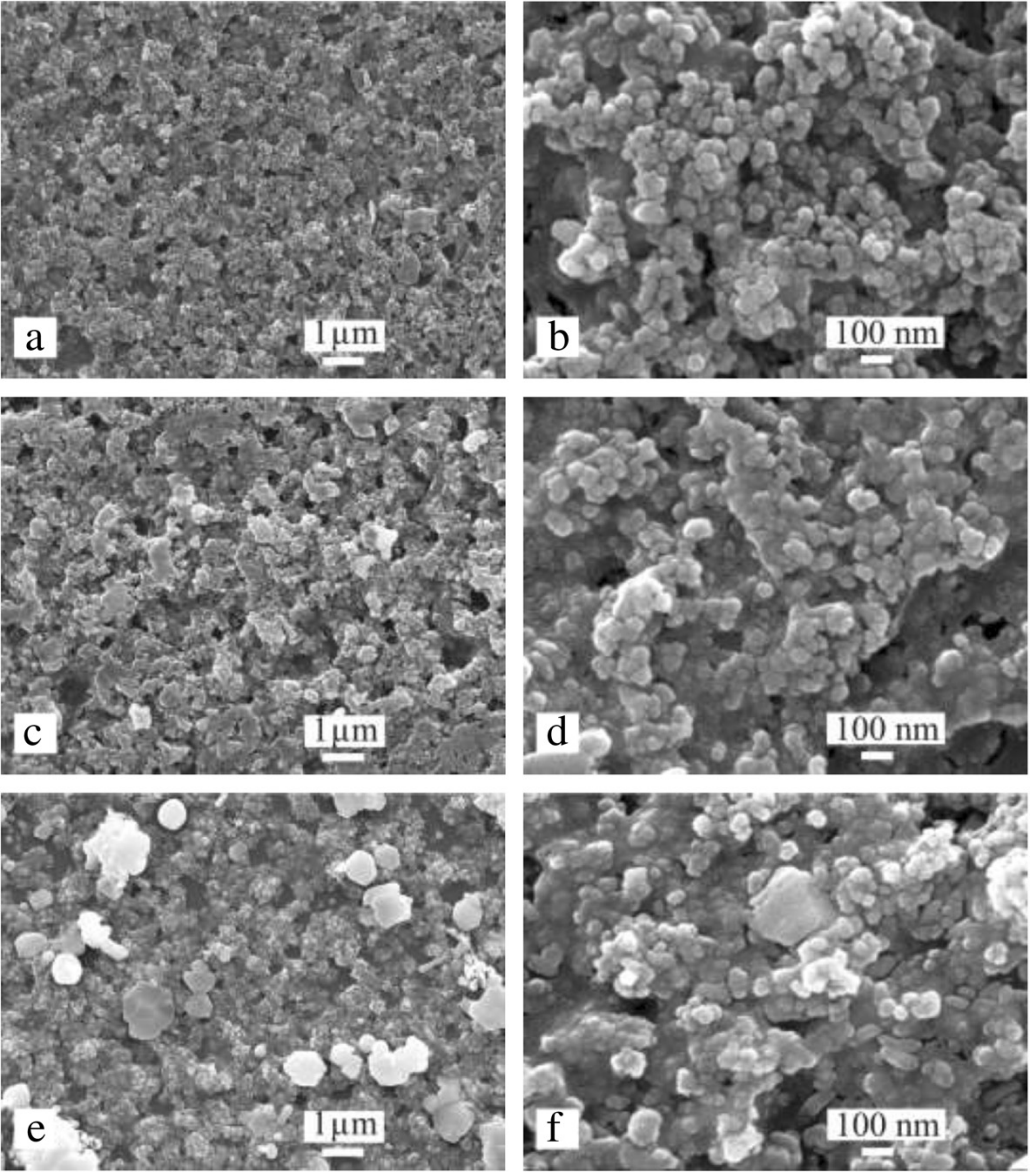
**Top-view SEM images at low and high magnifications.** TiO_2_/ITO **(a, b)**, Ag_2_S/TiO_2_/ITO **(c, d)**, and CdS(3)/Ag_2_S/TiO_2_/ITO **(e, f)** electrodes.

Figure 2 shows the typical surface SEM images of the prepared TiO_2_/ITO, Ag_2_S/TiO_2_/ITO, and CdS(3)/Ag_2_S/TiO_2_/ITO films. Figure 2a,b shows the top-view SEM images of the TiO_2_/ITO film. As shown in Figure 2a, the entire surface of the TiO_2_/ITO film is covered with TiO_2_ nanoparticles and is porous. Figure 2b shows a high magnification of the TiO_2_/ITO film, which clearly shows that the TiO_2_ nanoparticles with average diameters of approximately 25 nm are not uniformly distributed and the aggregation of TiO_2_ nanoparticles is observed. In addition, many nanoscale pinholes are distributed among the TiO_2_ nanoparticles on the surface of the TiO_2_/ITO film.

Figure 2c,d shows the top-view SEM images of the Ag_2_S/TiO_2_/ITO film. Figure 2c shows that, after the deposition of Ag_2_S, the number of the pinholes in the Ag_2_S/TiO_2_/ITO film is significantly reduced and the surface of the Ag_2_S/TiO_2_/ITO film became more flat compared to that of the TiO_2_/ITO film, which might be due to the filling of the Ag_2_S precursor solution in the low surface regions of the TiO_2_/ITO film. After the calcinations, more Ag_2_S nanocrystals that resulted from the Ag_2_S precursor solution aggregated in the low surface regions of the Ag_2_S/TiO_2_/ITO film and therefore improved the flatness of the film surface. This explanation can be supported by the higher magnification SEM image (Figure 2d) of the Ag_2_S/TiO_2_/ITO film. Figure 2d shows that the Ag_2_S nanocrystals appear to be fused together. In particular, the Ag_2_S nanocrystals in the low surface regions become fused together to form solid blocks.

Figure 2e,f shows the top-view SEM images of the CdS(3)/Ag_2_S/TiO_2_/ITO film. As shown in Figure 2e, after introduction of three cycles of CdS deposition, a large amount of CdS nanocrystals are deposited on the surface of the CdS(3)/Ag_2_S/TiO_2_/ITO film, and these deposited CdS nanocrystals are further fused together, which causes a large reduction in the number of pinholes. In addition, some lumps appear on the surface of the CdS(3)/Ag_2_S/TiO_2_/ITO film. Obviously, these lumps should be CdS that resulted from the residual CdS precursor solution on the surface of the film after the calcinations at 250°C. The corresponding high-magnification SEM image of the CdS(3)/Ag_2_S/TiO_2_/ITO film shown in Figure 2f further reveals that the CdS nanocrystals become fused together, which is similar to the case of Ag_2_S nanocrystals in the Ag_2_S/TiO_2_/ITO film.

**Figure 3:**
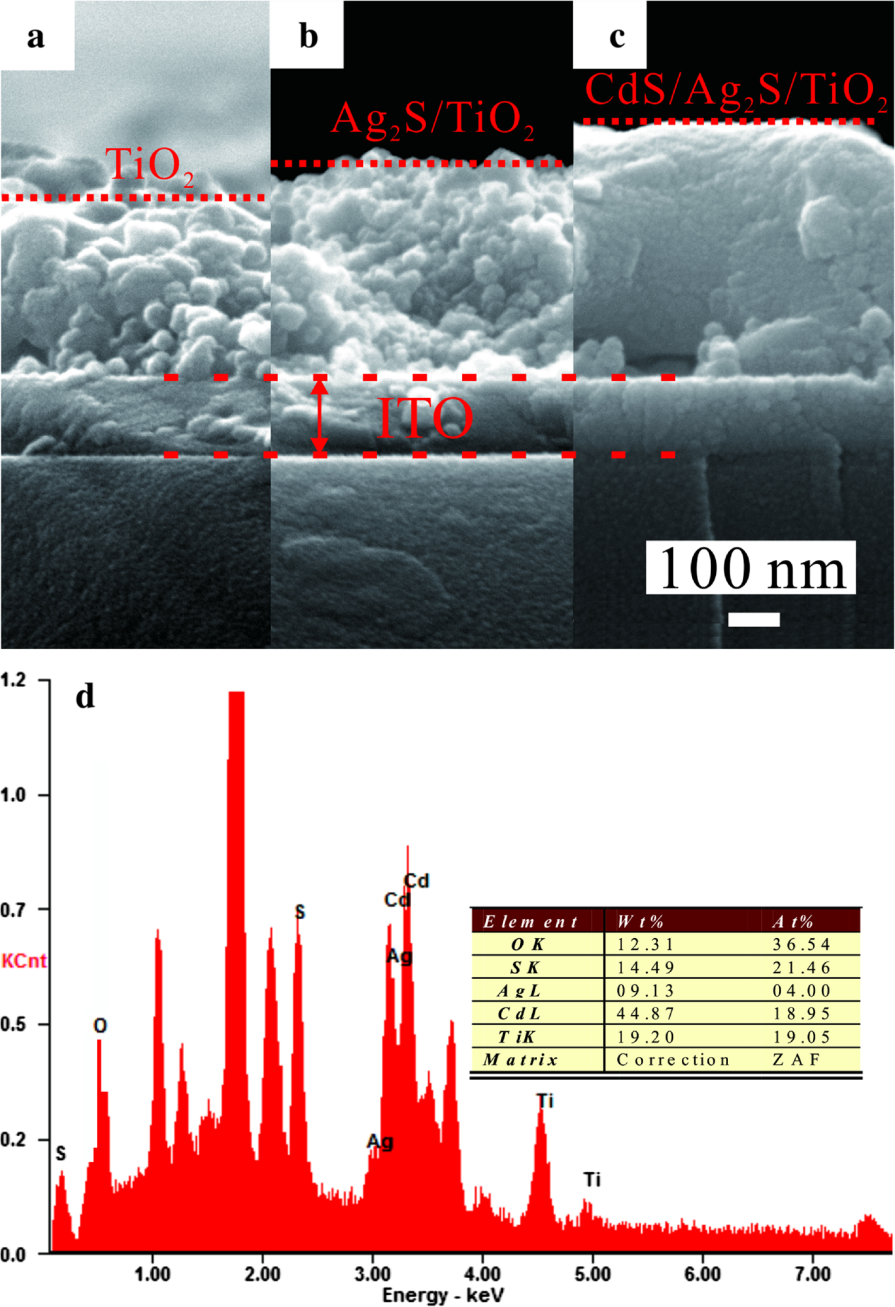
**SEM images and EDX spectra of electrodes.** Cross-sectional SEM images of **(a)** TiO_2_/ITO, **(b)** Ag_2_S/TiO_2_/ITO, and **(c)** CdS(1)/Ag_2_S/TiO_2_/ITO electrodes and **(d)** EDX spectra of the CdS(4)/Ag_2_S/TiO_2_/ITO electrode.

Figure 3a,b,c shows the cross-sectional SEM images of the TiO_2_/ITO, Ag_2_S/TiO_2_/ITO, and CdS(1)/Ag_2_S/TiO_2_/ITO films, respectively. From Figure 3a, it can be clearly seen that the average thickness of the TiO_2_ layer on the ITO substrate is about 350 nm. After the deposition of Ag_2_S nanocrystals, the average thickness (Figure 3b) of the Ag_2_S/TiO_2_ layer increases to about 420 nm. Moreover, it can be observed that, compared with the TiO_2_ layer, the Ag_2_S/TiO_2_ layer is more uniform and more compact, which is in agreement with the SEM results (Figure 2a,b,c,d). Similarly, after introduction of one cycle of CdS deposition, the CdS(1)/Ag_2_S/TiO_2_ layer results in an increased thickness of about 500 nm and improvements in the uniformity and compactness of the film. Figure 3d shows the EDX spectra on the region of the CdS(4)/Ag_2_S/TiO_2_/ITO film cross section. The EDX spectra reveals that the atomic composition of O, S, Ag, Cd, and Ti is 36.54%, 21.16%, 4.00%, 19.25%, and 19.05%, respectively. Therefore, the atomic ratio of Ti and O is close to 1:2, corresponding to the formation of TiO_2_. Besides, the difference between the atomic composition of S and that of Cd is 1.91%, which is about 0.5 times the atomic composition of Ag. Therefore, the EDX spectra and quantitative analysis results indicate that the TiO_2_, Ag_2_S, and CdS are formed.

**Figure 4:**
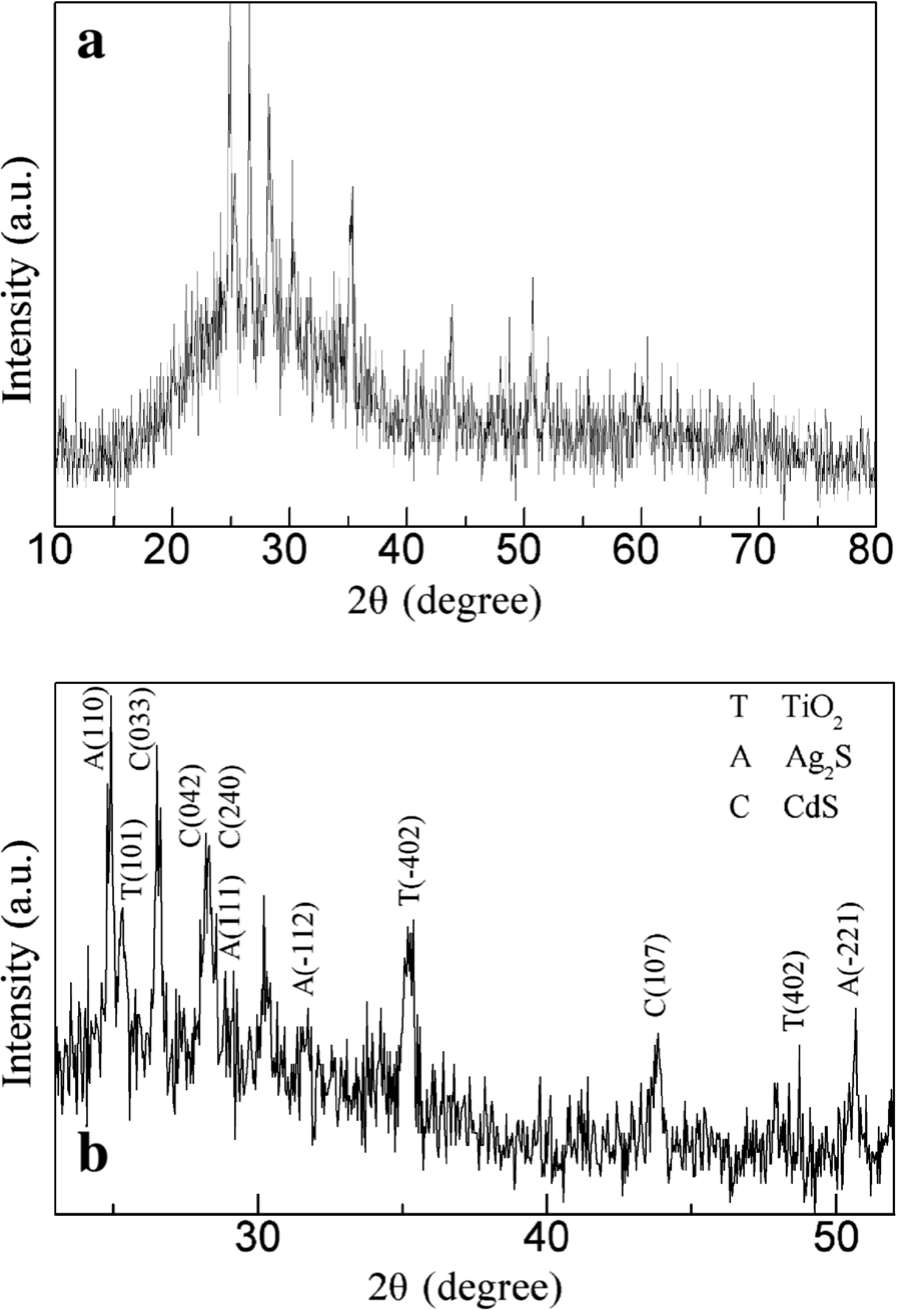
**XRD pattern of the CdS(1)/Ag**_**2**_**S/TiO**_**2**_**/ITO film.** XRD pattern of the CdS(1)/Ag_2_S/TiO_2_/ITO film **(a)** for 2*θ* between 10° and 80° and **(b)** for 2*θ* between 23° and 52°.

The crystalline phases of the prepared CdS(1)/Ag_2_S/TiO_2_/ITO film were characterized by XRD, as shown in Figure 4a. The magnified XRD patterns for 2*θ* between 23° and 52° is shown in Figure 4b. It can be seen that, for the prepared TiO_2_ layer, the diffraction peak at 2*θ* = 25.28° is observed, which can be indexed to the (101) lattice plane of anatase TiO_2_ (PDF# 21-1272). Besides, the diffraction peaks at 2*θ* = 35.30° and 48.68° are observed, which are ascribed to the (−402) and (402) lattice planes of the monoclinic phase of TiO_2_(B) (PDF# 46-1238), respectively. TiO_2_-B is a crystalline form of titania with a looser structure than anatase and rutile [39]. Therefore, the prepared TiO_2_ films are mixed phases of anatase and TiO_2_(B), which is similar to previous results [38,40]. It has been reported that, compared to a single-crystalline TiO_2_, a monocline and anatase bi-crystalline TiO_2_ is more conducive to the separation of photogenerated electrons and holes in the TiO_2_ and therefore has better electrical properties [41,42]. For the deposited Ag_2_S nanoparticles, the diffraction peaks at 2*θ* = 24.91°, 28.96°, 31.52°, and 50.73° are indexed to the (110), (111), (−112), and (−221) lattice planes of the monoclinic acanthite phase of Ag_2_S (α-Ag_2_S), respectively, which is in good agreement with the literature (PDF# 14-0072) [43,44]. Moreover, the 2*θ* peaks observed at 2*θ* = 26.48°, 28.15°, 28.28°, and 43.87° exhibit the formation of the orthorhombic phase of CdS (PDF# 47-1179) which correspond to the (033), (042), (240), and (107) lattice planes of reflections, respectively. These results further confirm the formation of Ag_2_S and CdS.

**Figure 5:**
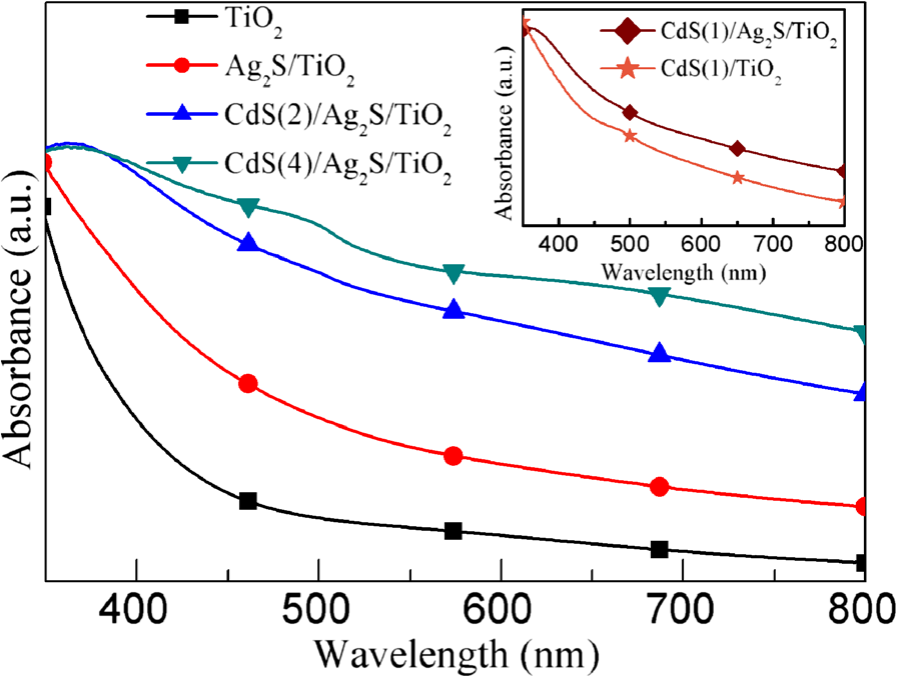
**UV-vis absorption spectra of TiO**_**2**_**/ITO, Ag**_**2**_**S/TiO**_**2**_**/ITO, and CdS( *n *)/Ag**_**2**_**S/TiO**_**2**_**/ITO.** UV-vis absorption spectra of the TiO_2_/ITO, Ag_2_S/TiO_2_**/**ITO, and CdS(*n*)/Ag_2_S/TiO_2_/ITO (*n* =2 and 4) in the dark and under simulated AM 1.5 G sunlight irradiation (100 mW cm^**−**2^).

Figure 5 shows the UV-visible (UV-Vis) absorption spectra of the TiO_2_/ITO, Ag_2_S/TiO_2_/ITO, and CdS(*n*)/Ag_2_S/TiO_2_/ITO (*n* = 2 and 4). As shown in Figure 5, barely TiO_2_/ITO film absorbs mainly the ultraviolet light with wavelengths smaller than 400 nm. However, when the Ag_2_S nanocrystals were deposited on the TiO_2_/ITO film, the light absorbance extends to the visible light region from 400 to 800 nm, which is apparently due to the characteristic absorption of Ag_2_S [33]. After the CdS deposition, the absorbance of the spectra of the CdS(*n*)/Ag_2_S/TiO_2_/ITO increases in the 350- to 800-nm wavelength region compared to that of the Ag_2_S/TiO_2_/ITO film, which may mainly result from the light absorption of CdS nanocrystals and the CdS/Ag_2_S/TiO_2_ composite system with a lower band gap than that of CdS due to the electron coupling between the CdS/TiO_2_ heterostructure [17,45-47]. In particular, for the CdS(4)/Ag_2_S/TiO_2_/ITO film, the absorption peak located at about 515 nm is observed, which is the characteristic absorption of the band gap of bulk CdS. Similar absorption peak is also observed in the CdS/ZnO/TNT film [17]. Therefore, these results of UV-Vis measurement further confirm the formation of Ag_2_S and CdS. Furthermore, due to the increased adsorption amount of CdS nanocrystals, the light absorbance of the spectra of the CdS(*n*)/Ag_2_S/TiO_2_/ITO films (*n* = 2 and 4) increases with the cycle number *n*. Additionally, for comparison, the absorption spectra of the CdS(*n*)/TiO_2_/ITO films were also measured and used to compare with that of the CdS(*n*)/Ag_2_S/TiO_2_/ITO films. As an example, the inset of Figure 5 shows the UV-Vis absorption spectra of the CdS(1)/Ag_2_S/TiO_2_/ITO and CdS(1)/TiO_2_/ITO films. It can be clearly seen that the UV-Vis absorption spectra of the CdS(1)/Ag_2_S/TiO_2_/ITO film is enhanced compared with the CdS(1)/TiO_2_/ITO film without Ag_2_S, which results from the light absorption of Ag_2_S nanocrystals. Apparently, the co-sensitized CdS(*n*)/Ag_2_S/TiO_2_/ITO films have enhancement effect in the light harvest, which is similar to the case of CdS and CuInS_2_ co-sensitized films [19].

**Figure 6:**
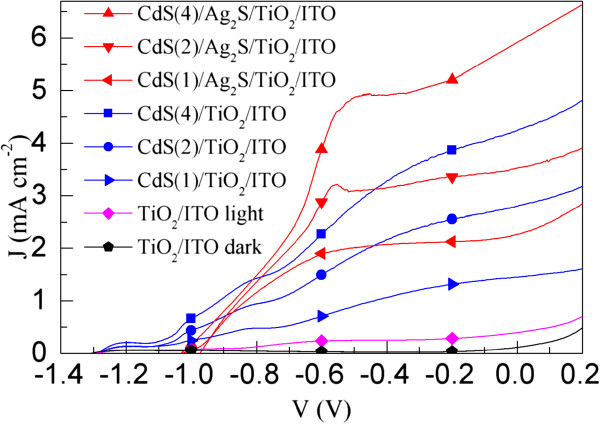
***J-V *** **characteristics of the TiO**_**2**_**/ITO, CdS(*n*)/TiO**_**2**_**/ITO, and CdS(*n*)/Ag**_**2**_**S/TiO**_**2**_**/ITO (*n = *** **1, 2, and 4) electrodes.**

**Figure 7:**
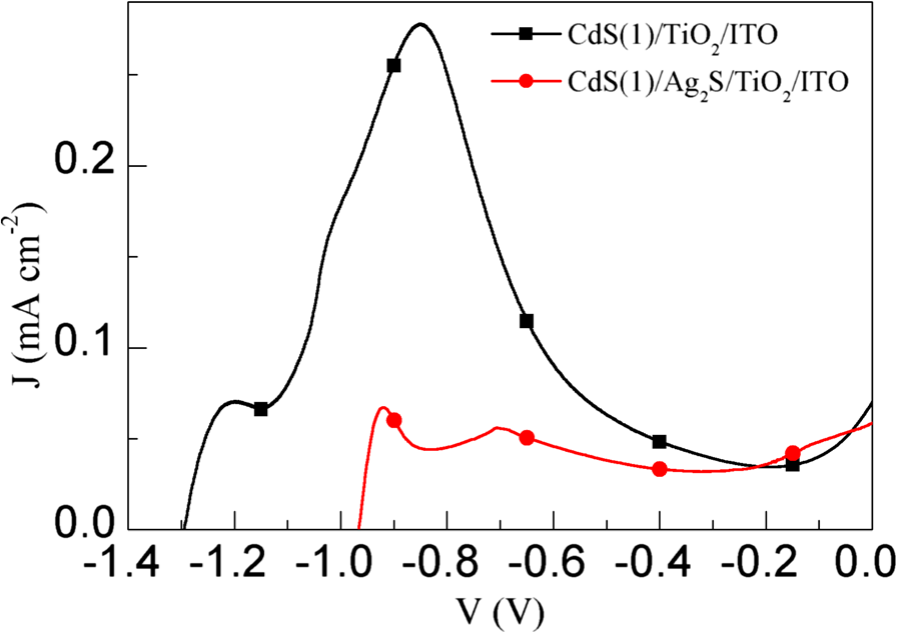
***J-V *** **characteristics of the CdS(1)/TiO**_**2**_**/ITO and CdS(1)/Ag**_**2**_**S/TiO**_**2**_**/ITO electrodes in the dark.**

To investigate the influence of Ag_2_S film on the photoelectrochemisty property of CdS-sensitized TiO_2_/ITO electrodes, potentiodynamic scans on the TiO_2_/ITO, CdS(*n*)/TiO_2_/ITO, and CdS(*n*)/Ag_2_S/TiO_2_/ITO (*n* = 1, 2, and 4) electrodes were performed vs. a saturated Ag/AgCl electrode with a scanning rate of 10 mV s^−1^ in the dark and under simulated AM 1.5 G sunlight irradiation (100 mW cm^−2^) as shown in Figure 6. It can be seen that the dark current density of the TiO_2_/ITO electrode is negligible. Under AM 1.5 G sunlight irradiation, all electrodes show pronounced photoresponses. The open-circuit voltage (*V*_oc_) of the TiO_2_/ITO electrode is 1.28 V versus the Ag/AgCl electrode, which is comparable with that of the TiO_2_ nanotube (TNT) electrode [17]. After the CdS deposition, the *V*_oc_ (1.29 V) of the CdS(*n*)/TiO_2_/ITO electrodes does not change obviously, which is very close to that (about 1.3 V) of reported CdS(*n*)/TNT electrodes [17]. However, after the deposition of Ag_2_S on the TiO_2_ film, the *V*_oc_ of the CdS(*n*)/Ag_2_S/TiO_2_/ITO electrodes decreases to about 1.0 V, which is in agreement with the obtained result from the Ag_2_S quantum dot-sensitized TNT electrodes [33]. The photocurrent density of the CdS(*n*)/TiO_2_/ITO electrodes increases with increasing deposition cycles, which can be attributed to the increased amount of CdS that can induce a higher photocurrent density [17,45]. For the same reason, a similar phenomenon is also observed in the CdS(*n*)/Ag_2_S/TiO_2_/ITO electrodes. The most important point is that, for a certain cycle *n* and an applied potential *V*, the photocurrent density of the CdS(*n*)/Ag_2_S/TiO_2_/ITO electrode is much higher than that of the CdS(*n*)/TiO_2_/ITO electrode when *V* > −0.8 V vs. Ag/AgCl. The highest photocurrent density at short circuit (*J*_sc_) achieved for the CdS(4)/Ag_2_S/TiO_2_/ITO electrode is 5.92 mA cm^−2^, which is much larger than that (4.67 mA cm^−2^) of the CdS(4)/TiO_2_/ITO electrode. There may be two reasons for the increased photocurrent density of the CdS(*n*)/Ag_2_S/TiO_2_/ITO electrodes. The first reason might be the increased absorbance of the CdS(*n*)/Ag_2_S/TiO_2_/ITO electrodes. As shown in the inset of Figure 5, compared to the CdS(*n*)/TiO_2_/ITO electrodes, the light absorbance of the CdS(*n*)/Ag_2_S/TiO_2_/ITO electrodes increased in the presence of the Ag_2_S nanocrystals, which would result in an increased photocurrent density. The other reason is probably reduced recombination of photoinjected electrons with redox ions from the electrolyte in the CdS(*n*)/Ag_2_S/TiO_2_/ITO electrodes. Similar to the case of CdS(*n*)/TNT electrodes, the charge recombination in the CdS(*n*)/TNT electrodes can be effectively suppressed through the interposition of an energy barrier layer, such as ZnO [17] or CuInS_2_[19], between the TNTs and electrolyte. In our case, the recombination of photogenerated electrons with redox ions from the electrolyte may be blocked by the Ag_2_S between the TiO_2_ film and CdS nanocrystals, which can be typically represented by the dark current of the electrodes [19,32]. Therefore, in order to investigate the influence of Ag_2_S on the charge recombination in the CdS(*n*)/TiO_2_/ITO electrodes, the *J*-*V* characteristics of the CdS(*n*)/TiO_2_/ITO and CdS(*n*)/Ag_2_S/TiO_2_/ITO electrodes in the dark are measured and compared. As an example, the *J*-*V* characteristics of the CdS(1)/TiO_2_/ITO and CdS(1)/Ag_2_S/TiO_2_/ITO electrodes in the dark are shown in Figure 7. It is surprising that, for the same applied potential *V*, the dark current density of the CdS(1)/TiO_2_/ITO electrode is much higher than that of CdS(1)/Ag_2_S/TiO_2_/ITO electrode, indicating that the incorporated Ag_2_S successfully reduced the charge recombination. Trying to explain this result would be an interesting work. According to common sense, the generated electrons in the CdS might not be effectively transferred to the Ag_2_S because the conduction band (CB) level of Ag_2_S is lower than that of TiO_2_ (Figure 1a). However, the efficient electron injection in Ag_2_S-sensitized TiO_2_ electrode has been experimentally found not only by us but also by others [23,24,33,34,48,49]. Since the rate of electron transfer from electron donor to electron acceptor depends on the energetic overlap of electron donor and acceptor [50,51], the reason for the electron transfer from α-Ag_2_S to TiO_2_ may be due to overlap of the electric states of α-Ag_2_S and TiO_2_ because the density of states of α-Ag_2_S can distribute in a wide energy range from −14 to 5 eV [52].

**Figure 8:**
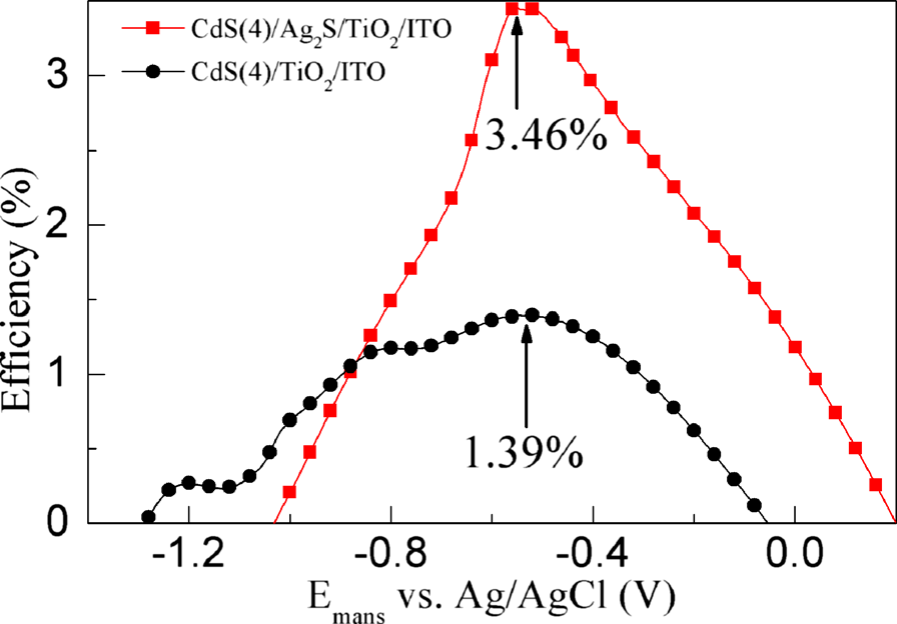
**The energy conversion efficiencies of the CdS(4)/TiO**_**2**_**/ITO and CdS(4)/Ag**_**2**_**S/TiO**_**2**_**/ITO electrodes.**

The light-chemical energy conversion efficiencies (*η*) of the CdS(4)/Ag_2_S/TiO_2_/ITO and CdS(4)/TiO_2_/ITO electrodes as a function of applied potential (vs. Ag/AgCl) under AM 1.5 G (100 mW/cm^−2^) illumination is calculated as *η* (%) = [(Total power output − Electric power input)/Light power input] × 100 (There are more details in [53]) [53], which is shown in Figure 8. It can be seen that an optimum energy conversion efficiency achieved by the CdS(4)/Ag_2_S/TiO_2_/ITO electrode is 3.46%, which is about 2.5 times that (1.39%) of the CdS(4)/TiO_2_/ITO electrode. It should be mentioned that the obtained *η* here is lower than that of the reported CdS-sensitized TNT/Ti electrode [17]. The main reason may be that the deposition amount of CdS on the TNT/Ti substrate with a length of 30 μm is more than that of CdS on the TiO_2_ nanocrystalline film with a thickness of about 350 nm. As discussed above, the improved *η* of the CdS(4)/TiO_2_/ITO electrode should be due to the increased light absorption and reduced charge recombination resulting from the deposition of Ag_2_S.

## Conclusions

To improve the efficiencies of CdS-sensitized TiO_2_/ITO electrodes, the CdS/Ag_2_S/TiO_2_/ITO electrodes were prepared by the interposition of Ag_2_S nanocrystalline film between the CdS and TiO_2_ nanocrystals as an energy barrier layer and a light absorber, in which both Ag_2_S and CdS nanocrystals were synthesized through a spin coating method of using a molecular-based precursor solution. The deposited Ag_2_S nanocrystals not only enhance the light absorption of the CdS-sensitized TiO_2_/ITO electrodes but also reduce the charge recombination, which resulted in the improved efficiencies of the CdS/Ag_2_S/TiO_2_/ITO electrodes. The maximum efficiency of the CdS(4)/Ag_2_S/TiO_2_/ITO electrode is 3.46%, which is about 2.5 times that (1.39%) of the CdS(4)/TiO_2_/ITO electrode without a Ag_2_S layer. Our research results indicate that the approach may provide a strategy to improve the efficiency of QSSCs.

## Competing interests

The authors declare that they have no competing interests.

## Authors' contributions

CC carried out the experiments, participated in the sequence alignment, and drafted the manuscript. YZ and CL performed the statistical analysis. FL participated in the device preparation and helped to discuss the experimental results. All authors read and approved the final manuscript.
